# Survivin as a mediator of stiffness-induced cell cycle progression and proliferation of vascular smooth muscle cells

**DOI:** 10.1063/5.0150532

**Published:** 2023-10-30

**Authors:** John C. Biber, Andra Sullivan, Joseph A. Brazzo, Yuna Heo, Bat-Ider Tumenbayar, Amanda Krajnik, Kerry E. Poppenberg, Vincent M. Tutino, Su-Jin Heo, John Kolega, Kwonmoo Lee, Yongho Bae

**Affiliations:** 1 Department of Pathology and Anatomical Sciences, Jacobs School of Medicine and Biomedical Sciences, University at Buffalo, Buffalo, New York 14203, USA; 2 Department of Biomedical Engineering, School of Engineering and Applied Sciences, University at Buffalo, Buffalo, New York 14260, USA; 3 Department of Pharmacology and Toxicology, Jacobs School of Medicine and Biomedical Sciences, University at Buffalo, Buffalo, New York 14203, USA; 4 Canon Stroke and Vascular Research Center, University at Buffalo, Buffalo, New York 14203, USA; 5 Department of Neurosurgery, Jacobs School of Medicine and Biomedical Sciences, University at Buffalo, Buffalo, New York 14203, USA; 6 Department of Orthopedic Surgery, Perelman School of Medicine, University of Pennsylvania, Philadelphia, Pennsylvania 19104, USA; 7 Vascular Biology Program, Boston Children's Hospital, Boston, Massachusetts 02115, USA

## Abstract

Stiffened arteries are a pathology of atherosclerosis, hypertension, and coronary artery disease and a key risk factor for cardiovascular disease events. The increased stiffness of arteries triggers a phenotypic switch, hypermigration, and hyperproliferation of vascular smooth muscle cells (VSMCs), leading to neointimal hyperplasia and accelerated neointima formation. However, the mechanism underlying this trigger remains unknown. Our analyses of whole-transcriptome microarray data from mouse VSMCs cultured on stiff hydrogels simulating arterial pathology identified 623 genes that were significantly and differentially expressed (360 upregulated and 263 downregulated) relative to expression in VSMCs cultured on soft hydrogels. Functional enrichment and gene network analyses revealed that these stiffness-sensitive genes are linked to cell cycle progression and proliferation. Importantly, we found that survivin, an inhibitor of apoptosis protein, mediates stiffness-dependent cell cycle progression and proliferation as determined by gene network and pathway analyses, RT-qPCR, immunoblotting, and cell proliferation assays. Furthermore, we found that inhibition of cell cycle progression did not reduce survivin expression, suggesting that survivin functions as an upstream regulator of cell cycle progression and proliferation in response to ECM stiffness. Mechanistically, we found that the stiffness signal is mechanotransduced via the FAK-E2F1 signaling axis to regulate survivin expression, establishing a regulatory pathway for how the stiffness of the cellular microenvironment affects VSMC behaviors. Overall, our findings indicate that survivin is necessary for VSMC cycling and proliferation and plays a role in regulating stiffness-responsive phenotypes.

## I. INTRODUCTION

Arterial stiffening contributes to the development and progression of a variety of cardiovascular diseases.^1–9^ The change in arterial stiffness affects the biophysical input to resident vascular smooth muscle cells (VSMCs), which constitute the majority of a biomechanically active and dynamic cell layer in the media of arteries. An increase in arterial stiffness triggers VSMCs to transition from a contractile (or differentiated) state to a synthetic (or dedifferentiated) state, in which they aberrantly migrate, proliferate, and produce extracellular matrix (ECM). This results in pathological neointima formation and further arterial stiffening.^8–11^ The stiffness of the ECM surrounding VSMCs thus regulates cardiovascular biology with implications for aging^12^ and Hutchinson–Gilford progeria syndrome^13^ as well as atherosclerosis,^5,14^ hypertension,^15,16^ coronary artery diseases,^17^ and fibrosis.^18^

To explore how ECM stiffness affects VSMC phenotype and function, we and others have used fibronectin- or collagen-coated deformable polyacrylamide hydrogels to create culture matrices mimicking normal (healthy) and pathological (diseased) stiffnesses. Studies with this system showed that ECM stiffness affects cell phenotype,^19,20^ cell cycle progression and proliferation,^4,7,21^ migration,^22–24^ cellular stiffness and traction force,^7,21,25,26^ cell–cell adhesion,^21^ and ECM synthesis.^5,12^ However, the signaling pathways regulating the response of VSMCs to ECM stiffness are yet to be fully defined.

There is evidence that VSMC responses to vascular injury and stiffening involve survivin^27–29^ (also known as Birc5 [baculoviral inhibitor of apoptosis (IAP) repeat-containing 5]), a member of the IAP family. Survivin was initially identified as an antiapoptotic protein in cancer and later shown to regulate cancer cell migration and proliferation.^30,31^ Survivin is expressed at a low level in healthy adult tissue but is rapidly upregulated in response to vascular injury, atherosclerosis, and hypertension (conditions in which arteries stiffen^4,5,7^) in animal models.^27,28,32^ Moreover, survivin is highly upregulated in proliferating VSMCs in the neointima and media in human atherosclerotic plaques and stenotic vein grafts.^28^ Interestingly, overexpression of a dominant-negative survivin mutant reduces neointima formation in rabbits,^27^ suggesting that survivin is a regulator of these effects.

Survivin induction after vascular injury correlates with the expression of cell proliferation genes downstream of focal adhesion kinase (FAK), which activates Rac to promote stiffness-sensitive cell cycle progression and proliferation of VSMCs.^7^ Furthermore, inhibition of FAK^33^ reduces survivin levels in other cell types. Because FAK is activated at sites of vascular injury and in VSMCs cultured on stiff hydrogels,^4,7,21^ we hypothesized that FAK contributes to the mechanotransduction of ECM stiffness to induce VSMC responses mediated by survivin. The objective of the present study was to identify the regulatory pathway through which the stiffness of the cellular microenvironment affects VSMC behavior. Our findings suggest that ECM stiffness coordinates with survivin and focal adhesion biology in an integrated mechanobiochemical system to control the cell cycle progression and proliferation of VSMCs.

## II. RESULTS

### A. ECM stiffness affects the phenotype of VSMCs

Increases in arterial and ECM stiffness contribute to phenotypic changes of VSMCs. We investigated the impact of ECM stiffness on cellular phenotypes by culturing mouse (m) or human (h) VSMCs on fibronectin-coated soft, medium, and stiff hydrogels (5.60 ± 2.45, 10.03 ± 2.49, and 21.05 ± 3.07 kPa, respectively, measured by atomic force microscopy) (Fig. S1). The soft and stiff hydrogels reflect the elastic moduli of healthy and injured/diseased mouse arteries,^4,5,12,34–36^ respectively. With this stiffness-tunable system, our transcriptome data analysis, detailed in Sec. II B of the results and Sec. I of the methods, identified phenotype markers^20,37–40^ for both contractile and synthetic VSMCs (Fig. S2 and Table S1**)**. The contractile markers included Kv1.5 (*Kcna5*), calmodulin 1 (*Calm1*), adipocyte enhancer binding protein 1 (*Aebp1*), smooth muscle myosin heavy chain (*Myh11*), myocardin (*Myocd*), leiomodin 1 (*Lmod1*), caldesmon 1 (*Cald1*), smoothelin (*Smtn*), smooth muscle actin (*Acta2*), transgelin (*Tagln*), and calponin (*Cnn1*) [Fig. S2(a)]. The synthetic markers included osteopontin (*Spp1*), retinol-binding protein 1 (*Rbp1*), galectin 3 (*Lgals3*), fibronectin 1 (*Fn1*), vimentin (*Vim*), Krüppel-like factor 4 (*Klf4*), nonmuscle myosin heavy chain B (*Myh10*), tropomyosin 4 (*Tpm4*), collagen type I alpha 2 chain (*Col1a2*), and collagen type I alpha 1 chain (*Col1a1*) [Fig. S2(b)]. Although the expression of more than half of these marker genes, as summarized in Table S1, did not change significantly in response to substrate stiffness, expression of *Acta2*, *Tagln*, *Cnn1*, *Spp1*, *Rbp1*, *Lgals3*, and *Col1a1* changed significantly. To experimentally validate our findings from the transcriptome analysis, we performed immunoblotting analysis using hVSMCs cultured on both soft and stiff hydrogels. Our data show there was an upregulation of the contractile markers smoothelin [Fig. S2(c)] and Myh11 [Fig. S2(d)] in cells cultured on soft hydrogels. Conversely, smooth muscle actin [Fig. S2(e)] and calponin [Fig. S2(f)], also contractile markers, along with synthetic markers Myh10 [Fig. S2(f)] and osteopontin [Fig. S2(g)] were upregulated in cells cultured on stiff hydrogels. Furthermore, our recent study demonstrated that stiff ECM increases the production of collagen and fibronectin, both of which are synthetic markers, to levels comparable to those observed in hVSMCs grown on the soft hydrogel.^41^ The presence of smoothelin and Myh11, considered strong contractile markers,^42–45^ indicates that VSMCs cultured on soft hydrogels have a contractile phenotype. However, the level of smooth muscle actin, the least specific contractile marker,^19^ was significantly upregulated on stiff hydrogels. Conversely, the upregulation of Myh10^46^ and osteopontin^47^ on stiff hydrogels indicates that high stiffness promotes a synthetic phenotype. These findings demonstrate that, within our hydrogel system, ECM stiffness selectively modulates gene and protein expression associated with VSMC phenotype.

### B. Whole-transcriptome analyses identify the stiffness-sensitive transcriptome of VSMCs

**FIG. 1.**
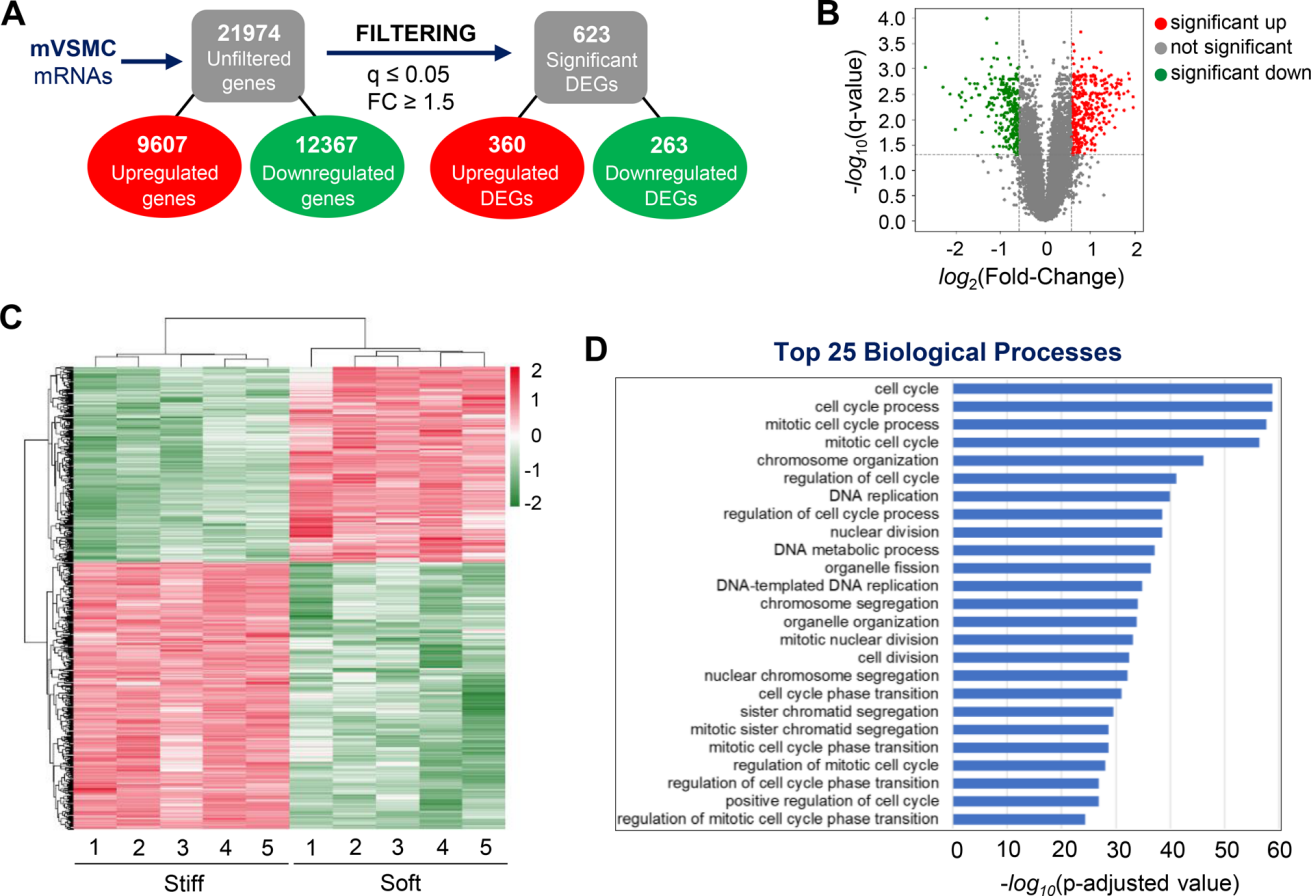
Whole-transcriptome analyses show the stiffness-sensitive transcriptome of VSMCs. (a) Reductions in data magnitude by applying significance thresholds to the raw microarray [absolute fold change (FC) ≥ 1.5 and *q*-value ≤ 0.05]. (b) Volcano plots display the distributions of all detected transcripts, represented as single dots that are not statistically different (gray), significantly upregulated (red), or significantly downregulated from data sequencing results. The *y* axis represents each gene's −log_10_(*q*-value), whereas the *x* axis represents their log_2_(fold change). (c) Heat maps display the Z-scores of the 623 DEGs. (d) Histograms present the top 25 biological processes enriched for 623 DEGs in the dataset.

In addition to impacting the phenotype of VSMCs modulated by ECM stiffness, an increase in ECM stiffness has been demonstrated to induce the cell cycle progression and proliferation of VSMCs.^4,7,21^ To further identify molecular targets involved in these processes, we examined the impact of ECM stiffness on the global transcriptome landscape of VSMCs *in vitro*. A whole-transcriptome microarray analysis was performed using mRNA samples from mVSMCs cultured for 24 h on fibronectin-coated soft or stiff hydrogels. To prevent cell–cell contact, VSMCs were seeded sparsely, and cell lysates were collected once the cell density reached approximately 70%–90% (after 24 h of cell culture). Our transcriptome data analysis identified a total of 21,974 genes (9607 upregulated and 12,367 downregulated) for which the expression in mVSMCs cultured on stiff hydrogels differed from that in cells on soft hydrogels [Fig. 1(a)]. These data were then filtered by absolute fold change and *q* value (absolute fold change ≥ 1.5 and q-value ≤ 0.05), resulting in a list of 623 statistically significant and differentially expressed genes (DEGs) [360 upregulated and 263 downregulated; Fig. 1(a) and Table S2]. The distribution of these DEGs against the total number of identified genes was plotted as the −log_10_(*q-*value) vs log_2_(fold change) values of each detected gene, with red color denoting significantly upregulated DEGs, green denoting significantly downregulated DEGs, and gray denoting genes with no significant change [Fig. 1(b)]. Additionally, the DEGs (Table S2) are depicted in a heat map, showing significant clustering between mVSMCs on soft and stiff hydrogels [Fig. 1(c)]. The heat map not only confirms the consistency of the Z-score signs among replicate samples for each DEG, indicating directional expression changes, but also highlights the reliability of the data.

We conducted a gene ontology (GO)-based functional enrichment analysis to identify biological processes associated with the upregulated DEGs. Our analysis revealed that the enriched biological processes were primarily related to the regulation of cell cycle progression, including cell cycle (GO:0007049), cell cycle process (GO:0022402), mitotic cell cycle process (GO:1903047), and mitotic cell cycle (GO:0000278) [Fig. 1(d)]. These GO findings confirmed that mVSMCs on stiff hydrogels express a significant number of genes that are known to affect multiple processes crucial to cell cycle progression and proliferation.

### C. Network analysis identifies survivin as a stiffness-sensitive mediator of cell cycle progression

**FIG. 2.**
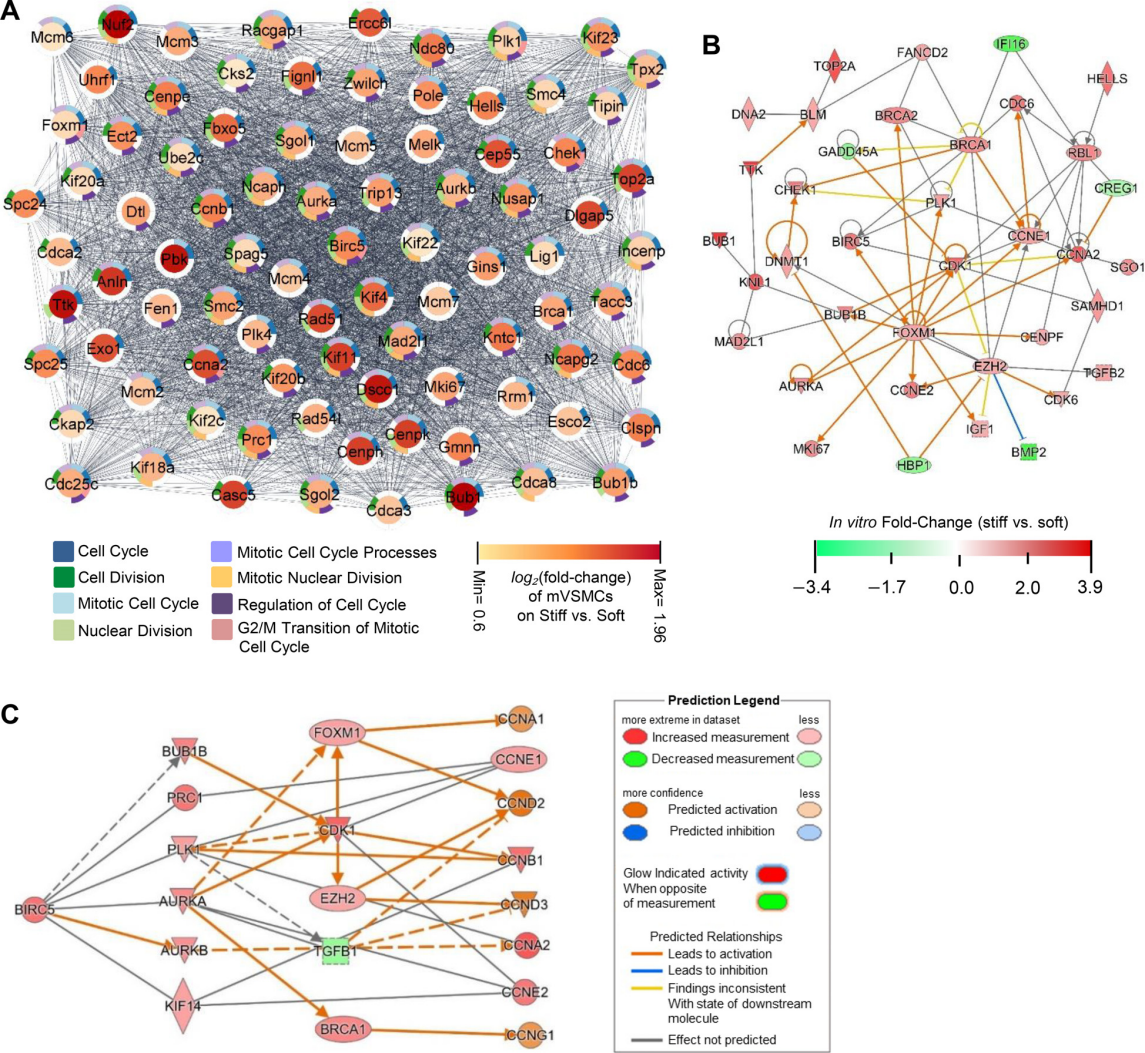
Network analysis identifies survivin (Birc5) as a potential stiffness-mediated regulator of cell cycle progression. (a) String network generated using the highly clustered 87 DEGs that are associated with cell cycle progression, with node color representing log_2_(fold change) values and border colors indicating DEG memberships to GO biological process categories. (b) Network diagram providing an overview of the molecular interactions related to Birc5 within the activated cell cycle progression function (Z-score = 2.124) in cardiovascular disease. (c) A predictive model for Birc5 regulation of cyclins. Network diagram presenting downstream targets of Birc5 (including cyclins) and potential intermediate molecules, with node color and intensity representing the observed expression levels or predicted activation states based on the microarray data.

To identify key regulators involved in stiffness-induced cell cycle progression, we used Cytoscape's String application to construct a network of 623 DEGs based on their functional associations or protein–protein interactions. Subsequently, we employed Cytoscape's MCODE (Molecular Complex Detection) plugin to identify highly interconnected regions in the network. Our analysis using MCODE revealed that 110 DEGs (nodes) clustered through 5658 connections (edges), and of these 110 DEGs, 87 genes that connected through 3583 edges were associated with cell cycle progression [Fig. 2(a)]. Notably, *Birc5*, which encodes survivin, was identified as one of the highly connected DEGs in the cluster, with 103 interactions with other genes as listed in Table S3. Moreover, we found that survivin was significantly upregulated and was linked to all of the cell cycle progression-associated biological processes highlighted in this network [Fig. 2(a)].

Previous studies showed an upregulation of survivin in response to cardiovascular conditions associated with arterial stiffening.^27–29^ Using ingenuity pathway analysis (IPA), we identified DEGs that interact with *Birc5* and are associated with both cell cycle progression (with a Z-score of 2.124; a Z-score of ≥ 2 is considered significant^48^) and cardiovascular disease. Figure 2(b) shows a predicted overview of the molecular interactions related to *Birc5* within the activated cell cycle progression function in cardiovascular disease, identifying several key cell cycle components such as *Cdk1*, *Ccna2*, *Ccne1*, *Ccne2*, and *Plk1*, all of which are associated with cell proliferation.

To further elucidate the molecular pathway linking cell cycle-associated DEGs (such as *Cdk1*, *Ccna2*, *Ccne1*, *Ccne2,* and *Plk1*) through *Birc5,* we employed IPA's Path Explorer tool and generated a network by overlaying fold change values from the dataset [Fig. 2(c)]. Our computational analyses suggest that survivin is a key regulator of stiffness-induced cell cycle progression and proliferation of VSMCs.

### D. Survivin is stimulated by pathological ECM stiffness in VSMCs

**FIG. 3.**
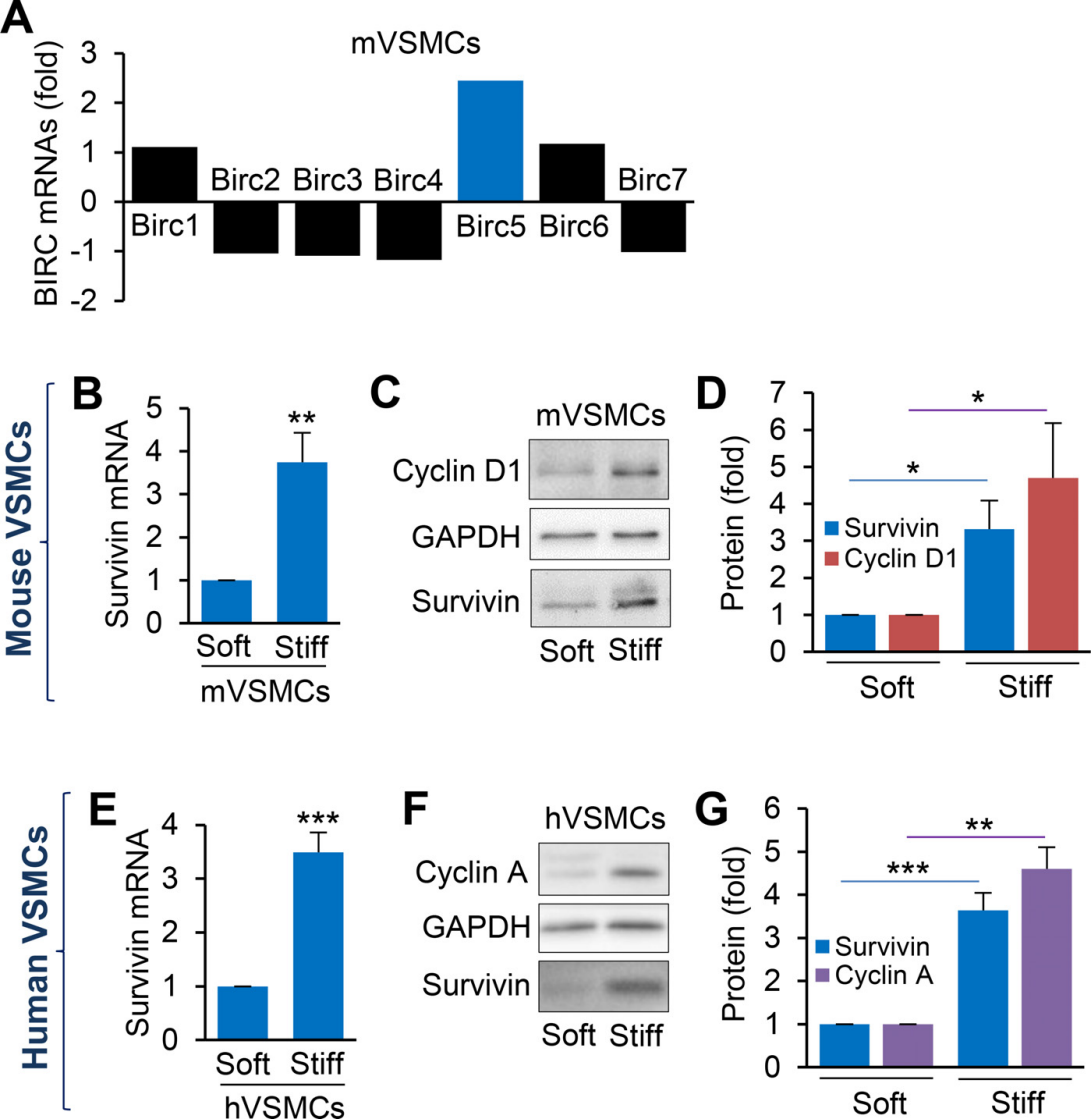
ECM stiffness modulates survivin expression in VSMCs. (a) Differential mRNA expression of baculovirus inhibitor of apoptosis repeat-containing (Birc) gene family from mouse VSMCs (mVSMCs) seeded on fibronectin-coated soft or stiff polyacrylamide hydrogels. G_0_-synchronized mVSMCs (b)–(d) or human (h)VSMCs (e)–(g) were seeded on soft or stiff hydrogels for 24 h. Survivin gene (b) and (e) and protein (c) and (f) expression was analyzed by RT-qPCR and immunoblotting, respectively. Levels of survivin mRNA were normalized to those measured in mVSMCs (b) and hVSMCs (e) on soft hydrogels; *n* = 5 (b) and *n* = 8 (e), independent experiments. Average survivin intensity in mVSMCs (c) and hVSMCs (f) was quantified using ImageJ and normalized to that in VSMCs on soft hydrogels. Representative immunoblot images from four independent biological replicates; GAPDH served as a loading control. Survivin protein levels normalized to that in mVSMCs (d) and hVSMCs (g) on soft hydrogels. Data are means + SEMs. ^*^p < 0.05, ^**^p < 0.01, and ^***^p < 0.001.

An analysis of the whole-transcriptome datasets for IAP family members other than Birc5 (survivin) identified *Naip1* (NLR family, apoptosis inhibitory protein 1 [*Birc1*]), *Birc2*, *Birc3, Xiap* (X-linked IAP [*Birc4*]), *Birc6*, and *Birc7*. However, only the expression of *Birc5* mRNA was significantly increased in mVSMCs grown on stiff hydrogels [2.5-fold compared to that in cells grown on soft hydrogels; Fig. 3(a)]. The increases in survivin levels were confirmed with real-time quantitative PCR (RT-qPCR) and immunoblotting from mVSMCs [Figs. 3(b) and 3(c)] and hVSMCs [Figs. 3(e) and 3(f) and S3(a) and S3(b)] cultured on soft and stiff hydrogels. Furthermore, protein levels of cyclins D1 and A (major targets of ECM stiffness-mediated signaling for cell cycle progression^4,7,21^) were sensitive to ECM stiffness in mVSMCs [Figs. 3(c) and 3(d)] and hVSMCs [Figs. 3(f) and 3(g)], respectively. Together with the results described above from the functional enrichment and network analyses (Fig. 2), these data suggest that stiffness-sensitive expression of survivin is associated with cell cycle progression.

### E. Survivin modulates stiffness-mediated cell cycling, proliferation, and cell spreading

**FIG. 4.**
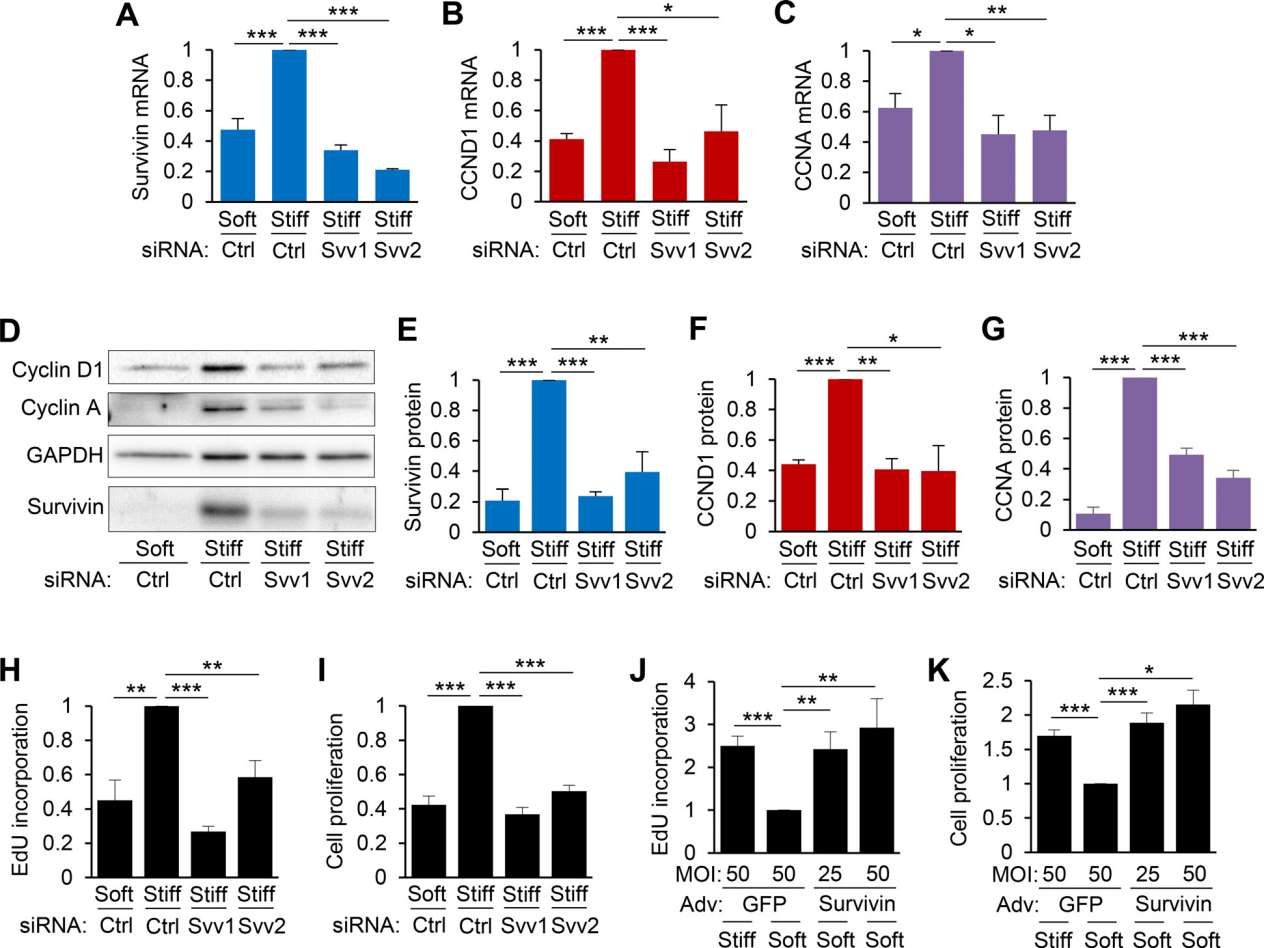
Survivin is required for stiffness-mediated cell cycle progression and proliferation. (a)–(g) hVSMCs were transfected with control siRNA or siRNAs to survivin [#1 and #2], synchronized to G_0_ via serum starvation, and plated on soft or stiff hydrogels with 10% FBS for 24 h. Total cell lysates were analyzed by RT-qPCR (a)–(c) or immunoblotting (d)–(g) to determine mRNA and protein levels of survivin (a) and (e), cyclin D1 [CCND1; (b) and (f)], and cyclin A [CCNA; (c) and (g)]. Expression levels were normalized to those in hVSMCs treated with control siRNA on stiff hydrogels. *n* = 4 (a), *n* = 3 (b), *n* = 3 (c), *n* = 5 (e), *n* = 3 (f), and *n* = 5 (g), independent experiments. S-phase entry and cell proliferation with survivin knockdown (h)–(i) and overexpression (j)–(k) were assessed by EdU incorporation (h) and (j) and cell counting (i) and (k), respectively, and normalized to values from hVSMCs treated with control siRNA on stiff hydrogels (h)–(i) or infected with GFP adenovirus (Adv) on soft hydrogels **(**j)–(k). *n* = 5 (h), *n* = 3 (i), *n* = 4 (j), and *n* = 4 (k), independent experiments. Data are means + SEMs. ^*^p < 0.05, ^**^p < 0.01, and ^***^p < 0.001.

We next examined if survivin expression affects cell cycle progression and proliferation. We transfected hVSMCs with either survivin-targeting siRNAs or a non-targeting negative control siRNA before culturing them on soft or stiff hydrogels. The targeted survivin siRNAs reduced survivin mRNA [Fig. 4(a)] and protein [Figs. 4(d) and 4(e)] expression by 70%–90% in hVSMCs cultured on stiff hydrogels compared to that in cells with control siRNAs on stiff hydrogels. Furthermore, knockdown of survivin decreased the stiffness-mediated induction of cyclin D1 [*CCDN1*; Figs. 4(b) and 4(f)] and cyclin A [*CCNA*; Figs. 4(c) and 4(g)] compared to expression in cells treated with control siRNAs on stiff hydrogels. Accordingly, S-phase entry and cell proliferation were reduced by survivin siRNAs in cells cultured on stiff hydrogels as determined by the incorporation of EdU (5-ethynyl-2′-deoxyuridine) [Fig. 4(h)] and cell counting [Fig. 4(i)], respectively. Similarly, blocking the induction of survivin with YM155 (a pharmacological agent that inhibits survivin expression^49^) in cells cultured on stiff hydrogels reduced expression of *CCND1*, *CCNA*, and *CCNB* (cyclin B) mRNA (Fig. S4). Interestingly, S-phase entry [Fig. 4(j)] and cell proliferation [Fig. 4(k)] were triggered in hVSMCs plated on soft hydrogels with adenovirus-mediated overexpression of survivin relative to that in hVSMCs infected with a control virus (for GFP expression) on soft hydrogels. These data demonstrate that survivin mediates the effects of ECM stiffness on cell cycle progression and proliferation.

We additionally assessed whether survivin overexpression on a stiff hydrogel further contributes to increased cell cycle progression and proliferation. To explore this, we cultured survivin-overexpressing VSMCs on stiff hydrogels for 24 h and evaluated cell proliferation. Our results show that survivin overexpression did not increase cell proliferation further when cells were cultured on stiff hydrogels (GFP-stiff vs Survivin-stiff; Fig. S5). Thus, it appears that the expression of survivin induced by stiff ECM is sufficient to promote cell proliferation, without the need for further survivin overexpression.

We further assessed the influence of ECM stiffness on VSMC morphology. For this, VSMCs were cultured on soft and stiff hydrogels for 24 h, fixed, stained with phalloidin, mounted with anti-fade medium containing DAPI (4′,6-diamidino-2-phenylindole), and imaged with a fluorescence microscope [Fig. S6(a)]. We found that increased ECM stiffness promoted cell spreading, as indicated by an increase in both area [Fig. S6(b)] and perimeter [Fig. S6(c)] and a decrease in circularity [Fig. S6(d)]. Conversely, VSMCs cultured on soft ECM showed reduced cell spreading [Figs. S6(b) and S6(c)]. These findings provide strong evidence that ECM stiffness affects VSMC morphology. We next asked whether survivin overexpression in VSMCs cultured on the soft hydrogel would phenocopy the cellular morphology and spreading observed on the stiff hydrogel [Fig. S6(e)]. To explore this, VSMCs infected with adenoviruses encoding either GFP or survivin were cultured on the soft and stiff hydrogels for 24 h. Survivin overexpression promoted modest cell spreading, as demonstrated by increased cell area [Fig. S6(f)] and perimeter [Fig. S6(g)] along with decreased circularity [Fig. S6(h)]. Additionally, our recent findings demonstrated that survivin overexpression increases intracellular stiffness in VSMCs cultured on soft hydrogels.^41^ Furthermore, survivin overexpression increases the production of collagen, fibronectin, and lysyl oxidase to levels comparable to those observed in cells grown on stiff hydrogels.^41^ Collectively, these findings suggest that survivin, in response to stiffness, orchestrates the morphological and mechanical behaviors of VSMCs while also modulating cell cycle progression and proliferation.

### F. Survivin is an upstream regulator of cell cycle progression as a function of ECM stiffness

**FIG. 5.**
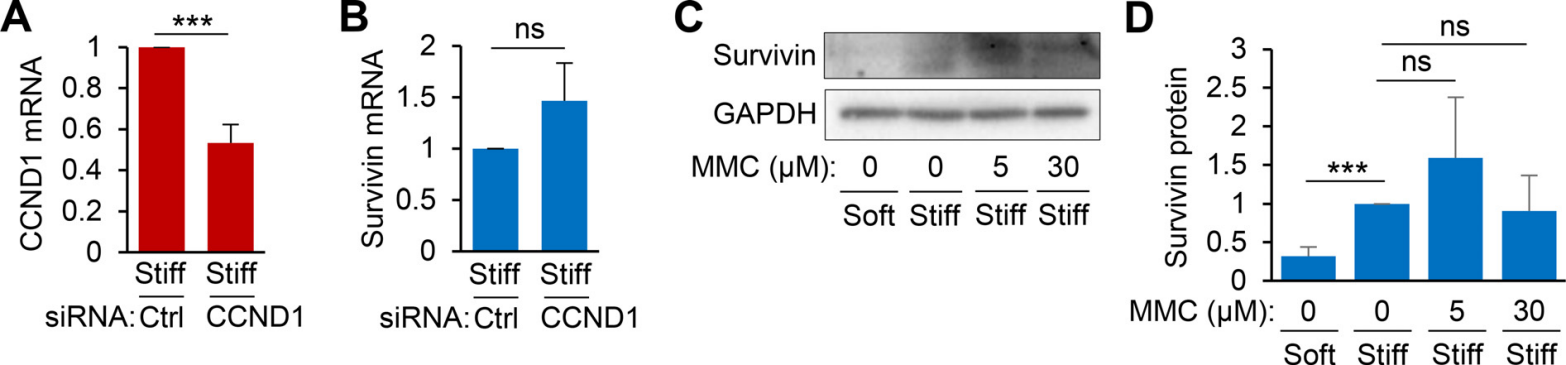
Survivin is an upstream regulator of stiffness-induced cell cycle progression. (a) and (b) hVSMCs were transfected with control siRNA or siRNAs to cyclin D1, synchronized to G_0_ via serum starvation, and plated on stiff hydrogels with 10% FBS for 24 h. Total cell lysates were analyzed by RT-qPCR to determine mRNA levels of cyclin D1 (CCND1) (a) and survivin (b). Expression levels were normalized to those in hVSMCs treated with control siRNA on stiff hydrogels. *n* = 3, independent experiments. (c) G_0_-synchronized hVSMCs were treated with either 5 or 30 *μ*M mitomycin C (MMC) and then cultured on soft or stiff hydrogels for 24 h. Survivin induction was analyzed by immunoblotting. Survivin protein levels were normalized to those in hVSMCs treated with DMSO on stiff hydrogels (d). *n* = 4–8, independent experiments.

To support the notion that survivin acts upstream of stiffness-induced cell cycle progression and proliferation, we examined if inhibition of cyclin D1 (an early G_1_ cell cycle regulator) affects survivin expression. To achieve this, we transfected VSMCs with cyclin D1 siRNA to transiently inhibit cyclin D1 expression and cultured the cells on hydrogels for 24 h. The RT-qPCR analysis showed that knockdown of cyclin D1 expression did not reduce survivin mRNA expression [Figs. 5(a) and 5(b)].

Additionally, we treated VSMCs with mitomycin C, as previously described,^50^ to globally inhibit cell proliferation. We then cultured the cells on soft or stiff hydrogels for 24 h. The immunoblotting analysis showed that 5 *μ*M mitomycin C treatment increased survivin expression modestly (but not significantly), whereas 30 *μ*M mitomycin C treatment did not decrease survivin expression [Figs. 5(c) and 5(d)]. This result, combined with the cyclin D1 knockdown data, further strengthens the notion that survivin functions as an upstream regulator of cell cycle progression and proliferation in response to ECM stiffness.

### G. FAK regulates stiffness-mediated survivin expression and cell cycling

**FIG. 6.**
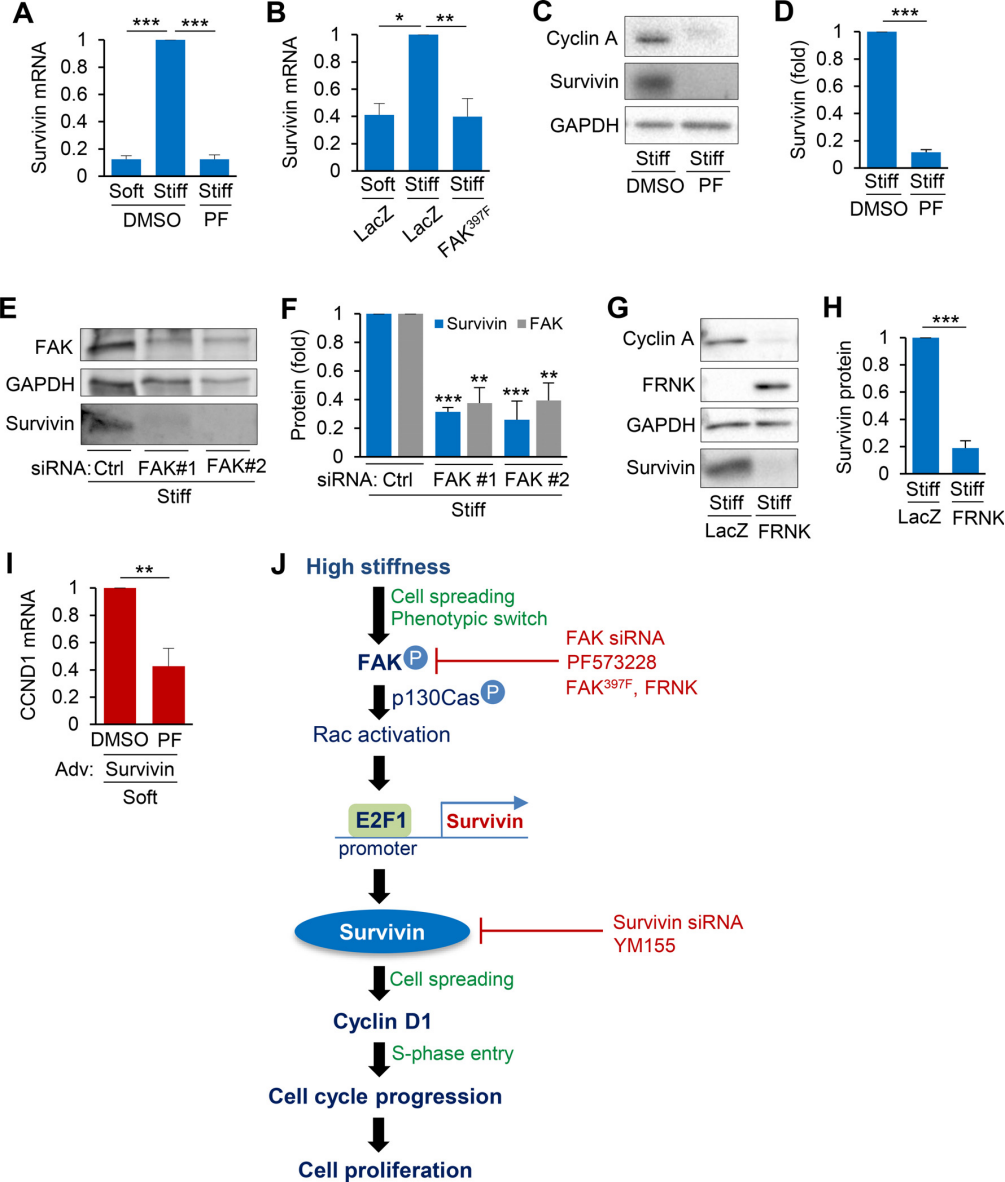
FAK regulates stiffness-mediated survivin expression. G_0_-synchronized hVSMCs treated with FAK inhibitor PF573228 (PF) in DMSO were seeded on fibronectin-coated soft or stiff hydrogels for 24 h. Survivin mRNA (a) and protein (c) and (d) expression were analyzed by RT-qPCR and immunoblotting, respectively; levels were normalized to those in hVSMCs treated with DMSO on stiff hydrogels; *n* = 3 (a) and *n* = 5 (c) and (d), independent experiments. (b) hVSMCs infected with adenoviruses encoding LacZ or FAK397F were seeded on soft or stiff hydrogels with 10% FBS for 24 h. Survivin mRNA expression was analyzed by RT-qPCR; levels were normalized to those in hVSMCs infected with LacZ on stiff hydrogels. *n* = 8, independent experiments. (e) and (f) G_0_-synchronized hVSMCs were transfected with control siRNA or siRNAs to FAK [#1 and #2], serum-starved, and plated on stiff hydrogels with 10% FBS for 24 h. FAK and survivin levels in total cell lysates were analyzed by immunoblotting; levels were normalized to those in hVSMCs treated with control siRNA on stiff hydrogels. *n* = 3, independent experiments. (g) and (h) hVSMCs infected with adenoviruses encoding LacZ or FRNK were plated on stiff hydrogels for 24 h. Protein expression levels were analyzed by immunoblotting. *n* = 4, independent experiments. (i) Survivin overexpressing hVSMCs treated with PF in DMSO were plated on soft hydrogels for 24 h. Cyclin mRNA expression was analyzed by RT-qPCR. *n* = 4, independent experiments. (j) Model of signal transduction for stiffness-mediated cell cycle progression. Data are means + SEMs. ^*^p < 0.05, ^**^p < 0.01, and ^***^p < 0.001.

Previous studies showed that FAK phosphorylation increases with vascular injury and stiffness-induced cell cycle progression and proliferation^4,7,21^ and that survivin induction after vascular injury correlates with the expression of cell cycle genes downstream of FAK.^7^ Therefore, we investigated whether FAK is responsible for stiffness-mediated survivin expression. Treatment of hVSMCs on stiff hydrogels with PF573228 (PF; FAK-specific inhibitor) markedly reduced the levels of survivin mRNA [Fig. 6(a)], and survivin and cyclin A protein [Figs. 6(c) and 6(d)]. These effects were confirmed by a reduction in survivin expression in hVSMCs infected with adenoviruses encoding FAK397 (a nonphosphorylatable form of FAK) compared to that in cells expressing the LacZ control [Fig. 6(b)]. Additionally, siRNA-mediated knockdown of FAK [Figs. 6(e) and 6(f)] and adenovirus-mediated overexpression of FAK-related non-kinase (FRNK) [Figs. 6(g) and 6(h)] similarly reduced survivin [Figs. 6(e)–6(h)] and cyclin A [Fig. 6(g)] levels in hVSMCs cultured on stiff hydrogels compared to that in controls. We further assessed whether FAK inhibition affects cell cycle progression in survivin overexpressing VSMCs cultured on soft hydrogels. To investigate this, survivin-overexpressing VSMCs treated with PF573228 were cultured on soft hydrogels for 24 h. Our results demonstrate that FAK inhibition reduced cyclin D1 mRNA levels in survivin-overexpressing cells [Fig. 6(i)], suggesting that FAK activity is essential for survivin-mediated cell cycle progression.

FAK inhibition reduces the stiffness-dependent activation of Rac.^4,7,21,51^ Furthermore, FAK-Rac signaling targets a transcriptional factor E2F1, which binds to the survivin promotor and promotes survivin transcription.^52,53^ Therefore, we investigated whether FAK mediates stiffness-dependent survivin expression through E2F1. To examine this relationship, we treated VSMCs with 10 *μ*M PF573228 (or dimethyl sulfoxide [DMSO] as the control) and cultured them on hydrogels. Western blotting analysis revealed that E2F1 expression was increased by the stiff ECM and that the inhibition FAK decreased the stiffness-dependent expression of E2F1 (Fig. S7). Collectively, our results suggest that the mechanotransduction of ECM stiffness activates FAK-E2F1 signaling, leading to the upregulation of survivin and cyclin D1, consequently triggering cell cycle progression and proliferation in VSMCs [Fig. 6(j)].

## III. DISCUSSION

Stiffness-mediated remodeling of the vascular wall involves complex interactions between the local microenvironment and VSMCs. Moreover, the phenotypic switch, accelerated cell cycle progression, and proliferation of VSMCs appear to both cause and result from the arterial stiffening process. Indeed, our data show that ECM stiffness affects the phenotype, morphology, cell cycle progression, and proliferation of VSMCs. To identify the molecular mechanism by which stiffness affects VSMC cycling and proliferation, we applied integrative genome-wide analyses to databases from cellular models of arterial stiffening and cell proliferation. The evidence suggests that survivin is the molecular linchpin by which ECM stiffness mediates pathological VSMC behaviors.

Survivin is known as a critical regulator of mitosis and cytokinesis during cancer cell division. Overexpression of survivin in the nuclei of cancer cells promotes the G_1_–S cell cycle transition by releasing p21 (known as a cyclin-dependent kinase [Cdk] inhibitor 1) from Cdk4 to activate the cyclin D1/Cdk4 complex, leading to the phosphorylation of the protein retinoblastoma.^54,55^ Furthermore, high ECM stiffness promotes this cyclin D1/Cdk4-dependent phosphorylation of retinoblastoma protein (Rb).^4,7,21^ We showed that downregulation of survivin reduces the stiffness-induced upregulation of cyclin proteins, including cyclin D1 (expressed in early G_1_ phase), cyclin A (expressed in S phase), and cyclin B (expressed in G_2_/M phase), as well as cell spreading in VSMCs grown on stiff hydrogels. Furthermore, ectopic expression of survivin in VSMCs cultured on soft hydrogels was sufficient to promote cell cycling and proliferation and modestly increase cell spreading. Interestingly, our findings revealed that inhibition of cell cycle progression does not impact survivin expression, indicating that survivin acts as an upstream regulator of cell cycle progression and proliferation in response to ECM stiffness. Together with previous studies, our findings suggest a novel mechanism by which the upregulation of survivin in VSMCs in response to ECM stiffness stimulates mechanosensitive cell cycling and proliferation.

The findings presented here further expand on our work showing that stiffness-sensitive cell cycle progression and proliferation are associated with FAK, p130Cas, and Rac.^4,7,21^ In our previous studies, we demonstrated that ECM stiffness increases FAK and p130Cas phosphorylation and Rac activation, and that inhibiting these proteins reduces intracellular stiffness and stiffness-mediated cell cycle progression, suggesting that cells sense ECM stiffness through FAK-p130Cas-Rac-dependent signaling.^4,7,21^ Furthermore, the inhibition of FAK interfered with stiffness-mediated expression of survivin, which involved FAK-Rac targeting of E2F1 (Fig. S7); E2F1 binds to the survivin promotor^53^ and induces survivin expression.^52^ Furthermore, FAK and Rac activate the transcription factor STAT3,^56,57^ which also regulates the survivin promoter.^58^ However, further studies are needed to detail how FAK-Rac signaling triggers survivin transcription.

The protein YAP, activated by FAK,^59^ also binds to the survivin promoter, driving survivin transcription,^60^ and plays a role in the mechanotransduction of ECM stiffness into cell cycle progression and proliferation.^61^ Interestingly, a recent study showed that verteporfin, a benzoporphyrin derivative, inhibits YAP/TAZ nuclear localization, leading to their degradation and a subsequent reduction in survivin expression in gastric cancer cells.^62^ Another study demonstrated that interfering with YAP nuclear localization with cerivastatin (a hydroxymethylglutaryl-CoA reductase inhibitor) reduces the expression of cyclins A and B, thereby regulating cell cycle progression, potentially independent of survivin.^63^ Therefore, YAP's role in stiffness- and survivin-mediated cell cycle progression should be examined to further detail the underlying molecular mechanisms.

In summary, we demonstrated that ECM stiffness signals an increase in survivin expression in VSMCs through FAK-E2F1 signaling, driving cell cycle progression and proliferation. Additionally, in line with our previous research, we showed that the FAK-p130Cas-Rac pathway modulates stiffness-induced cyclin D1 expression.^4,7,21,51^ Collectively, these findings represent a novel mechanism [Fig. 6(j)] through which mechanical changes in the microenvironment trigger a pathological switch of VSMCs from a contractile phenotype to a synthetic phenotype. Importantly, these findings also open up potential targets for therapies addressing vascular and cardiovascular diseases characterized by aberrant cell proliferation and arterial stiffening.

## IV. METHODS

### A. Cell culture

Primary hVSMCs (catalog number [Cat. No.] 354-05a, Cell Applications, Inc.) were maintained in ≤90% Dulbecco's modified Eagle's medium (DMEM) supplemented with 1 mM sodium pyruvate, 2% MEM amino acid solution, 50 *μ*g/ml gentamicin solution, 1% penicillin–streptomycin solution, and 10% fetal bovine serum (FBS). Primary mVSMCs were prepared from explant cultures of thoracic aortae from 2-month-old male C57BL/6 mice and maintained at ≤90% confluency in low-glucose DMEM–F12 medium (1:1) supplemented with 10% FBS, 2 mM glutamine, 25 *μ*M HEPES, 1% penicillin–streptomycin solution, and 50 *μ*g/ml gentamicin solution. Both cell types were maintained in 10% CO_2_ at 37 °C and used before passage 5. To synchronize VSMCs to the G_0_ cell cycle phase, cultures that were near confluence were incubated in serum-free DMEM containing 1 mg/ml heat-inactivated, fatty-acid-free bovine serum albumin (BSA) for 48 h. The starved cells were then treated with 0.05% trypsin–EDTA, centrifuged, resuspended, and plated on soft or stiff hydrogels with fresh medium containing 10% FBS for 24 h.

### B. siRNA transfection

hVSMCs were transfected with 200 nM siRNAs targeting survivin, FAK, or cyclin D1 (or negative control siRNA [cat no. AM4636]) by using Lipofectamine 2000 or 3000 reagents in Opti-MEM as previously described.^7,51^ After 4–5 h of siRNA transfection, cells were serum starved in fresh DMEM containing 1 mg/ml BSA for 43–44 h. All siRNA-based experiments were performed 72 h after transfection. The following survivin (*BIRC5*), FAK (*PTK2*), and cyclin D1 (*CCND1*) siRNAs were obtained from Ambion: survivin siRNA #1 (ID no. 121294), 5′-CCACUUCCAGGGUUUAUUCtt-3′; survivin siRNA #2 (ID no. 121295), 5′-GCCAUUCUAAGUCAUUGGGtt-3′; FAK siRNA #1 (ID no. 157448), 5′-CCUAGCAGACUUUAACCAAtt-3′; FAK siRNA #2 (ID no. 61352), 5′-GGCAUGGAGAUGCUACUGAtt-3′; and cyclin D1 siRNA (ID no. 42828), 5′-GGAGAACAAACAGAUCAUCtt-3′.

### C. Adenovirus infection

hVMSCs were first incubated in DMEM containing 1 mg/ml BSA for 8 − 9 h. Cells were then incubated for 20–24 h with adenoviruses encoding wild-type survivin (Cat. No. 1611, Vector Biolabs; multiplicity of infection [MOI], 25 and 50) or FRNK (a gift from the Assoian Laboratory; MOI, 600); adenoviruses encoding GFP (Cat. No. 1060, Vector Biolabs; MOI, 50) or LacZ (a gift from the Assoian Laboratory; MOI, 600) were used as the respective experimental controls.

### D. Drug treatment

Serum-starved hVSMCs were plated on fibronectin-coated soft or stiff hydrogels with medium containing 10% FBS and were treated with 0.1, 0.5, or 2 *μ*M YM155 (survivin inhibitor; Cat. No. 11490, Cayman Chemical), 10 *μ*M PF573228 (FAK inhibitor; Cat. No. 14924, Cayman Chemical), 5 or 30 *μ*M mitomycin C (proliferation inhibitor; Cat. No. BML-GR311-0002, Enzo Life Sciences), or DMSO (vehicle control).

### E. Preparation of stiffness-tunable hydrogels

The soft (2–8 kPa) and stiff (18–24 kPa) polyacrylamide hydrogels^7,64^ approximate the physiological stiffness of a healthy mouse femoral artery and an artery after vascular injury and atherosclerosis,^4,5^ respectively. The protocol for generating stiffness-tunable polyacrylamide hydrogels was previously described.^51,64^ Briefly, glass coverslips were etched homogenously with a 0.1 M NaOH solution for 3 min and then treated with 3-(trimethoxysilyl)propyl methacrylate (Cat. No. 440159, Sigma-Aldrich) to introduce amine groups to cross-link with the polyacrylamide hydrogel. Hydrogels of different stiffness were prepared by changing the ratio of 40% acrylamide to 1% bis-acrylamide in a mixed solution with sterilized water, ammonium persulfate (Cat. No. A3678, Sigma-Aldrich), TEMED (Cat. No. J63734. AC, Thermo Fisher Scientific), and an N-hydroxysuccinimide-fibronectin solution prepared by combining one part N-hydroxysuccinimide solution (Cat. No. A8060, Sigma-Aldrich; 1 mg/ml in DMSO) and nine parts fibronectin solution (Cat. No. 341631, Calbiochem; 100 *μ*l fibronectin at 1 *μ*g/*μ*l dissolved in 1.9 ml Tris base [pH 8.4 pH]). Finally, the fibronectin-coated hydrogels were extensively washed in Dulbecco's phosphate-buffered saline (DPBS) and water to remove unpolymerized polyacrylamide, and unreactive cross-linkers were blocked with 1 mg/ml BSA before VSMCs were seeded.

Hydrogel stiffness was measured by atomic force microscopy as previously described.^4,7,21^ Briefly, hydrogels were indented with a circular symmetric tip (Cat. No. QP-BioAC-Cl or Cat. No. qp-SCONT, NanoAndMore USA Corp). Atomic force microscopy in contact mode was applied to a hydrogel using a Park NX12 AFM system (Park Systems) mounted on a Nikon ECLIPSE Ti2 inverted microscope.

### F. RNA isolation and RT-qPCR

hVSMCs and mVSMCs cultured on soft or stiff hydrogels for 24 h were treated with TRIzol reagent to extract total RNA. The RNA was reverse transcribed and analyzed by RT-qPCR as previously described.^51^ TaqMan probes (Invitrogen) were used for survivin (Mm00599749_m1 for mouse mRNA and Hs04194392_s1 for human mRNA), GAPDH (Mm99999915_g1 for mouse mRNA and Hs02786624_g1 for human mRNA), cyclin D1 (Hs00765553_m1), cyclin A (Hs00171105_m1), cyclin B (Hs00259126_m1), and E2F1 (Hs00153451_m1). The relative change in mRNA expression for each target mouse or human gene was determined by the comparative threshold cycle method using the gene for GAPDH as the reference.

### G. Protein extraction, immunoblotting, and immunostaining

As previously described,^51^ total cell lysates were collected from hVSMCs or mVSMCs cultured on soft or stiff hydrogels by incubating the hydrogels face down for 2 min at room temperature on 5× sample buffer (250 mM Tris [pH 6.8], 10% SDS, 50% glycerol, 0.02% bromophenol blue, and 10 mM 2-mercaptoethanol). Equal amounts of extracted protein were fractionated on reducing 8–12% SDS-polyacrylamide gels, and the fractioned proteins were subsequently transferred electrophoretically onto polyvinylidene fluoride membranes via the Trans-Blot Turbo Transfer System (Bio-Rad). These membranes were blocked in 6% nonfat milk for 1.5 h at room temperature and then probed with antibodies against survivin (Cat. No. NB500-201, Novus Biologicals; 1:200), cyclin D1 (Cat. No. sc-20044, Santa Cruz Biotechnology; 1:200), cyclin A (a gift from the Assoian Laboratory,^65^ 1:500), FAK (Cat. No. 39–6500, Invitrogen; 1:500), smoothelin (Cat. No. SC-376902, Santa Cruz Biotechnology; 1:200), osteopontin (Cat. No. SC-21742, Santa Cruz Biotechnology; 1:200), Myh10/myosin IIB (Cat. No. 3404, Cell Signaling Technology; 1:1000), Myh11 (Cat. No. SC-6965, Santa Cruz Biotechnology; 1:200), α-smooth muscle actin (Cat. No. 48938, Cell Signaling Technology; 1:500), calponin 1 (Cat. No. ab700, Abcam; 1:200), or GAPDH (Cat. No. 60004-1-Ig or 10494-1-AP, Proteintech; 1:5000). Antibody signals were detected using Clarity (Cat. No. 1705061, Bio-Rad) or Clarity Max (Cat. No. 1705062, Bio-Rad) Western ECL substrate.

For immunostaining, VSMCs cultured on hydrogels were fixed in 3.7% formaldehyde in DPBS for 1 h, permeabilized with 0.4% Triton X-100 for 30 min, and then blocked with 2% BSA and 0.2% Triton X-100 in DPBS for 1 h. Cells were incubated with Alexa Fluor 488-phalloidin (Cat. No. A12379, Invitrogen) for 1 h. Subsequently, cells were washed with DPBS containing 2% BSA and 0.2% Triton X-100, followed by a wash with sterile water before finally mounting with DAPI-containing mounting medium (Cat No. 17985–50, Electron Microscopy Sciences) for fluorescence microscopy. Fluorescence images of cells were acquired using a Leica DM 6B upright fluorescence microscope and analyzed using ImageJ.

### H. EdU incorporation and cell counting

Serum-starved hVSMCs treated with control or survivin siRNAs or infected with adenovirus encoding wild-type survivin or GFP were plated on fibronectin-coated stiff or soft hydrogels with 10% FBS and then incubated with 20 *μ*M EdU for 24 or 36 h. Cells were fixed in 3.7% formaldehyde and visualized using the Click-iT EdU Alexa Fluor 594 imaging kit (Cat. No. C10339, Invitrogen) according to the manufacturer's instructions. Nuclei were stained with DAPI, and coverslips were mounted on microscope slides. Three to eight fields of view were counted per coverslip to determine the percentage of hVSMCs with DAPI-stained nuclei that were positive for EdU.

For cell counting, six fields were imaged with the ZOE Fluorescent Cell Imager (Bio-Rad), and the nuclei were counted for each sample to determine the total number of nuclei. The total nuclei counts were compared among different experimental conditions to calculate the fold change ratio for each condition.

### I. Bioinformatics analysis

(1) Gene expression analysis: Differential gene expression analysis was performed on raw microarray data. Duplicate and blank (no name) gene entries were removed from data lists, and genes with nonsignificant differential expression values were filtered out before further analysis. For microarray data, DEGs were defined as genes having a ≥1.5-fold change and a Benjamini–Hochberg adjusted *p* value (*q*-value) of ≤0.05. Python's bioinfokit and R programming language's pheatmap packages were used to generate volcano plots and heat maps with hierarchical clustering, respectively. Euclidean distance as the similarity measure was used on normalized count data for hierarchical clustering of DEGs and samples.
(2) Functional enrichment analysis: A functional enrichment analysis was conducted using the g:GOSt tool in gProfiler (https://biit.cs.ut.ee/gprofiler/gost) on a list of 623 DEGs. The statistical domain scope of the analysis was limited to annotated genes, with a significance threshold set to the Benjamini–Hochberg algorithm for computing multiple-testing correction for *p* values acquired from Gene Ontology (GO). GO terms with an adjusted *p* value of ≤0.05 were considered significant, and the top 25 Biological Process GO terms were presented in histograms on a scale of −log_10_(adjusted *p* value). Cytoscape's String application was used to create a network of all gene interactions for the 623 DEGs. The resulting dataset was used to identify highly clustered regions in the network using Cytoscape's MCODE application, which resulted in 110 DEGs (nodes) and 5,658 connections (edges). The expression data for these DEGs were imported into the node table to indicate expression levels using log_2_(fold change) values and node color, indicating intensity. Nodes that did not display a relationship to any of the following Biological Process GO categories were filtered out: cell cycle (GO:0007049), regulation of cell cycle (GO:0051726), mitotic cell cycle (GO:0000278), mitotic cell cycle processes (GO:1903047), G_2_/M transition of mitotic cell cycle (GO:0000086), cell division (GO:0051301), nuclear division (GO:0000280), and mitotic nuclear division (GO:0140014). The resulting network contained 87 genes (nodes) and 3,583 connections (edges) [Fig. 2(a)].
(3) Network analysis: Ingenuity pathway analysis (IPA, Qiagen) was used to perform further bioinformatics analysis on the filtered microarray data. A core analysis was run on each of the datasets, which returned information on various mechanistic pathways and enriched functions based on the literature compiled in the Ingenuity Knowledge Base. The “Diseases and Functions” tool was used to identify molecules known to be involved in cell cycle progression within the *in vitro* dataset, and the “My Pathway” tool was subsequently used to display known relationships between *Birc5* and other genes within the cell cycle progression function. The Z-directional components of the expression analysis were based on the expression log ratio values. Functions with a Z-score of >2 were regarded as having significant activation, whereas those with a Z-score of <−2 were considered as having significant inhibition. The “Molecule Activity Predictor” tool was used to display gene expression levels via node color and intensity and to generate predicted activation states of molecules and interactions based on the results of the Core Analysis.

### J. Statistical analysis

Statistical significance was assessed for all the data using paired, two-tailed Student's *t* tests. The graphs present means + SEMs from the indicated number of independent experiments.

## SUPPLEMENTARY MATERIAL

See the supplementary material for three tables with microarray data and seven additional figures.

## Data Availability

The data that support the findings of this study are available from the corresponding author upon reasonable request.
